# Reference Echovirus 7 and 19 Genomes from Nigeria

**DOI:** 10.1128/MRA.01465-18

**Published:** 2018-12-06

**Authors:** T. O. C. Faleye, O. M. Adewumi, J. A. Adeniji

**Affiliations:** a Department of Virology, College of Medicine, Faculty of Basic Medical Sciences, University of Ibadan, Ibadan, Nigeria; b Department of Microbiology, Faculty of Science, Ekiti State University, Ado-Ekiti, Nigeria; c WHO National Polio Laboratory, University of Ibadan, Ibadan, Nigeria; University of Rochester School of Medicine and Dentistry

## Abstract

We describe the genomes of two echovirus isolates from Nigeria as reference enterovirus species B genomes for the region. These echovirus 7 and 19 genomes have 7,411 nucleotides (nt) and 7,426 nt and were recovered from sewage-contaminated water (in 2010) and an acute flaccid paralysis case (in 2014), respectively.

## ANNOUNCEMENT

Echoviruses belong to species B within the genus Enterovirus, family *Picornaviridae*, order *Picornavirales.* They have been recovered from clinical manifestations that range from respiratory disease to acute flaccid paralysis (1). The majority of the non-polio enteroviruses recovered in the rhabdomyosarcoma (RD) and L20B cell line-based algorithm recommended by the WHO (2) yield Enterovirus *B* (EV-B) members. Here, we describe the genome of two EV-Bs from Nigeria as references for the region.

The two isolates (E7 isolated from sewage-contaminated water in 2010 and E19 isolated from a child with acute flaccid paralysis in 2014) were initially cultured in the rhabdomyosarcoma (RD) cell line, produced a cytopathic effect, and were subsequently passaged twice in the cell line before further analysis. Thereafter, the RNA genomes were isolated using a total RNA extraction kit (Jena Bioscience, Jena, Germany). A Script cDNA synthesis kit (Jena Bioscience) was then used to convert the RNA genomes to cDNA as recommended by the manufacturer. The genomes were subsequently amplified in overlapping fragments of 2 to 3 kb using the Redload PCR kit (Jena Bioscience) and a combination of previously described primers (3–8). For each isolate, the overlapping genomic fragments were pooled and shipped to a commercial facility (MR DNA, TX, USA) where library preparation and NextGen sequencing were done. Library preparation was done using the Nextera DNA sample preparation kit (Illumina) following the manufacturer’s user guide. Sequencing was done paired end for 300 cycles using the HiSeq system (Illumina). Assembly was done using the Kiki Assembler v0.0.9.

For the E7 and E19 isolates, 3,478,802 and 3,458,346 reads were generated, respectively. The E7 and E19 genomes contain 7,411 and 7,426 nucleotides assembled from 2,469,874 (71%) and 2,873,503 (83.09%) reads, respectively. Both genomes have a G+C content of 47.8% and a single polyprotein (identified by aligning them with previously characterized and annotated EV-B genomes) with 2,194 and 2,196 amino acid residues, respectively. The E7 genome is most similar to that of isolate Env_2016_Sep_E-7a (GenBank accession number MG451805), another E7 recovered from sewage in the United Kingdom in 2016, but has been suggested to be of sub-Saharan African origin (9). The E19 genome, on the other hand, is most similar to that of isolate ETH_P3/E19_2016 (MF990292), an E19 recovered in April 2016 from the stool of a child in Ethiopia (10). Though EV-B genomic ends have been well characterized and the primers used in this study are based on conserved sequences (3–8), considering that the primers were used for amplification, the ends of the genomes might be of primer origin.

Here, we describe the genomes of two EV-Bs from Nigeria. These will serve as reference genomes for future EV-B genomic studies in the region.

### Data availability.

The genomes described have been deposited in GenBank with the accession numbers MH732737 and MH745407. The raw reads have also been deposited in the SRA with the BioProject numbers PRJNA497728 and PRJNA497733, respectively.
